# Mapping Breast Cancer Microenvironment Through Single-Cell Omics

**DOI:** 10.3389/fimmu.2022.868813

**Published:** 2022-04-20

**Authors:** Zhenya Tan, Chen Kan, Minqiong Sun, Fan Yang, Mandy Wong, Siying Wang, Hong Zheng

**Affiliations:** ^1^ Department of Pathophysiology, Anhui Medical University, Hefei, China; ^2^ Department of Biological Sciences, Georgia Institute of Technology, Atlanta, GA, United States

**Keywords:** breast cancer, microenvironment, single-cell RNA sequencing, single-cell omics, heterogeneity

## Abstract

Breast cancer development and progression rely not only on the proliferation of neoplastic cells but also on the significant heterogeneity in the surrounding tumor microenvironment. Its unique microenvironment, including tumor-infiltrating lymphocytes, complex myeloid cells, lipid-associated macrophages, cancer-associated fibroblasts (CAFs), and other molecules that promote the growth and migration of tumor cells, has been shown to play a crucial role in the occurrence, growth, and metastasis of breast cancer. However, a detailed understanding of the complex microenvironment in breast cancer remains largely unknown. The unique pattern of breast cancer microenvironment cells has been poorly studied, and neither has the supportive role of these cells in pathogenesis been assessed. Single-cell multiomics biotechnology, especially single-cell RNA sequencing (scRNA-seq) reveals single-cell expression levels at much higher resolution, finely dissecting the molecular characteristics of tumor microenvironment. Here, we review the recent literature on breast cancer microenvironment, focusing on scRNA-seq studies and analyzing heterogeneity and spatial location of different cells, including T and B cells, macrophages/monocytes, neutrophils, and stromal cells. This review aims to provide a more comprehensive perception of breast cancer microenvironment and annotation for their clinical classification, diagnosis, and treatment. Furthermore, we discuss the impact of novel single-cell omics technologies, such as abundant omics exploration strategies, multiomics conjoint analysis mode, and deep learning network architecture, on the future research of breast cancer immune microenvironment.

## Introduction

Breast cancer is the most frequent cancer and the leading cause of cancer-related death in women worldwide. Breast cancer progression is a complex process that coordinates the crosstalk between tumor cells and the components of tumor microenvironment (TME) (1). Breast TME as tumor components can dynamically program tumor growth (1). Tumor microenvironment generally includes immune cells, stromal cells, blood vessels, and extracellular matrix (ECM). Most of the stromal cells and immune cells experience some changes and play roles in both the suppression and progression of tumor (2, 3). In breast cancer, some TME components can modulate immune cells to counteract their intrinsic antitumor activity. For example, neutrophils exhibit tumor cytotoxicity during early disease stages, whereas in high burden tumor, they can be reprogramed to promote disease progression and dissemination (4). Therefore, breast TME is essential for the survival and immunosuppression of tumor cells.

Tumor environment supports tumor cell survival and evolution in the face of various tumor-adverse interventions (5), and destruction of the TME homeostasis can force tumor cell apoptosis and activate the T-cell-mediated cytotoxicity (6–8). TME can also modulate angiogenesis, cytokine secretions, and immune cell recruitment (9, 10).

Traditionally, biological experiments for TME analysis such as immunohistochemistry (IHC), immunofluorescence (IF), the emerging cytometry by time-of-flight (CyTOF) (11), or latest multiplexed ion beam imaging by time-of-flight (MIBI-TOF) (12) could only target certain cell populations preventing a holistic analysis of the highly heterogenous TME. However, the rise of single-cell omics in the past 10 years allowed us to understand the changes in cell populations, biochemical profile, and immune state of the TME during disease progression and partially addressed the shortcomings of purely biological assays.

**Figure 1:**
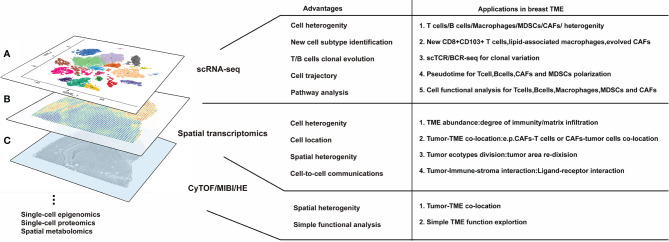
Several high-dimensional approaches for understanding breast tumor microenvironment (TME) composition and interaction. **(A)** The advantages of single-cell RNA sequencing. scRNA-seq is the most modern and popular technology for breast TME analysis, which is applicable for cell heterogeneity analysis and new cell subtype identification; moreover, scRNA-seq can be derived for T/B cell clonal evolution, cell trajectory, and pathway analysis in breast TME studies. **(B)** Meanwhile, the advantages of spatial transcriptomics are used to research cell orientation, tumor ecotypes, and cell-to-cell communication in breast TME. **(C)** The basal HE/CyTOF/MIBI are always used for visualization and assessment of breast TME; however, they have limited information for breast TME analysis. None of these methods can reach the three standards of single-cell level, high-throughput, and *in situ* reproducibility at the same time, so scRNAseq, spatial transcriptomics, or HE/CyTOF/MIBI are often combined to analyze the tumor microenvironment from multiple dimensions. Furthermore, a variety of omics methods (such as single-cell epigenomics, proteomics, and spatial metabolomics) can still be utilized for breast TME research but are more difficult to implement due to lack of evidence or technical limitations.

The emergence of these novel technologies explores a myriad of factors in the TME that were previously unattainable. FACS-based smart-seq2 or nanowell-based platforms explore cell alternative splicing (13, 14), while Sci-RNA-seq can detect rare cell populations such as cancer stem cell, circulating tumor cell, or rare immune cells (15, 16). Single-cell assay for transposase-accessible chromatin sequencing (scATAC-seq) and ChIP sequencing (scChIP-seq) epigenetically explore chromatin accessibility and transcriptional factor regulation (17, 18); whereas, scDNA-seq has become the most widely used assay to evaluate single-cell copy-number aberration (19). Meanwhile, the continuous design of integrated tools for single-cell omics not only detected cell heterogeneity but also extended analysis for transcription-based cell cloning aberration (20), cell traceability (21), cell-to-cell interaction (22, 23), rare cell resolution (24), and disease process simulation (25), giving us a deeper understanding of the intricate tumor malignancy. Therefore, this review summarizes the current state of the art on the analysis of breast cancer tumor microenvironment from single-cell omics (**Figure 1**). Altogether, we construct a comprehensive view of the TME and track the complex dynamic relationship between immune and stromal cells. Lastly, we discuss the near future research tendencies of single-cell omics and its impact in breast cancer research.

## The Plasticity of Breast Cancer Microenvironment

Surprisingly, preneoplastic cells do not significantly change the microenvironment for early malignant transformation and there is no difference of the breast microenvironment between preneoplastic and normal breast tissues (26). In fact, malignant cells are responsible for tumor microenvironment reprogramming likely caused by the exponential division of tumor cells and immune cell recruitment. Therefore, the microenvironment is constantly changing with tumor progression, from tiny tumor foci to palpable tumor mass. Early “indolent” tumor areas are dominated by infiltrating T cells and B cells with immune killing properties. However, progressive tumor regions require increased proliferation and an immunosuppressive milieu. Immune-suppressive T cells secrete IL-17 to recruit neutrophils and macrophages (27), which play an important role in higher myeloid cell infiltration and tumor metastasis in the clinical patients.

## The Heterogeneity of Lyphocytes in Breast TME

Classical prediction analysis of microarray 50 (PAM50) classifies breast cancer into luminal A, luminal B, HER2+, and basal-like subtypes according to the expression of estrogen receptor (ER), progesterone receptor (PR), and HER2 (28, 29). Regardless, clone aberration generates high heterogeneity even within each breast cancer subtype (30), so do with TME. Elham Azizi et al. used scDrop-seq to analyze CD45^+^ immune cell in 8 breast cancer patients and found that the immune cell subtypes were highly heterogeneous. T-cell fractions are the most abundant immune cells (21%–96%) in breast TME, followed by myeloid cells. Moreover, T cells in tumor and adjacent lesions had transcriptional similarities, which were significantly different from those in the peripheral circulatory system, indicating reprogramming of T cells by local primary tumors. Functionally, T cells were flexible in oxidative phosphorylation, IFN, TNF-a, TGF-b, IL-6/JAK/STAT, hypoxia, proinflammation, activation, and cytolytic effector pathways in local primary tumors. These reflected various differentiated and activated states in 32 T-cell clusters, but still immunogenic and exerting immune responses. Moreover, combined antigenic TCR stimulation and environmental factors reprogrammed similar biological function to form T-cell niches. For example, similar CD4^+^ T-cell populations exist in different breast cancer patients, such as homogenous population of Treg immunosuppressive cells prevalently present in all breast cancer subtypes. Interestingly, in addition to naturally Treg cells, traceability analysis found tumors could repolarize CD4^+^ conventional T cells to immunosuppressive Tregs (31). Whereas CD4^+^ and CD8^+^ T cells are widely dysfunctional, some representative CD8^+^ T-cell subgroups had significant immune suppressive activity (26, 32). These interact with Tregs and PD-L1^+^ tumor-associated macrophages (TAMs) to establish complex immunosuppressive niches (33). Therefore, the elimination of Tregs and PD-L1^+^ TAMs in the tumor may recover cytotoxic effect of T cells and enhance the effect of immune checkpoint blockade (ICB). Simultaneously, some T cells expressed less PD-1 but other immune checkpoints such as TIGIT and LAG3 which might prove novel ICB targets (34). Furthermore, a typical CD8^+^CD103^+^ tissue-resident memory cell population was also considered to be immunosuppressive. This T-cell subset seems to respond to immune checkpoint blockade (ICB) (35).

Interestingly, when comparing the immune microenvironment in different PAM50 subtypes, ER^−^ patients had the most Tregs, PD-L1^+^ TAMs, and PD-1 high CTLA-4^+^ CD38^+^ exhausted T cells, revealing that breast cancer microenvironment is remodeled by the endocrine system to promote immunosuppressive function. This also explained why ER^−^ patients are more likely to benefit from immunotherapy (33).

The specific role of B cells is still largely unknown in breast cancer and other cancers. It remains uncertain if B cells promote or restrict tumor growth. Hu et al. performed scRNA-seq-based BCR-seq analysis on B cells of breast cancer patients and showed that the B cell increased BCR diversity in the tumor, demonstrating the clone evolution and complex immunogenicity. The B-cell populations in the tumor were classified into 7 groups, including naïve B cells, IGM^+^CD27^+^ memory B cells, IGM^+^CD27^−^ atypical memory B cells, class-switched memory B cells, plasma cells, germinal center B cells, and CD14^+^ atypical B cells. Among these, memory B cells were the most abundant cell subgroup in the tumor, which differs from the enriched naïve B-cell population in the peripheral blood. Nevertheless, intratumoral B cells mainly showed potential immunogenicity and antigen presentation activity, and no specific cell population seemed to contribute directly to cytotoxic or immunosuppressive function. Hence, the specific function of B cells in the TME remained unclear (36). Recently, B cells were associated to ICB response in renal cell carcinoma (37), melanoma (38), and sarcoma (39). Sc-RNAseq analysis of B-cell function in breast cancer patients proved its respond to immune therapy and produced cascaded antibodies to activate cytotoxic T cells and amplify the ICB effect (40). Noteworthy, B cells in response to chemotherapy retained plasticity, as chemotherapeutic TME signals, such as complement signaling and inflammatory response, generate ICOSL^+^ B cells. These boost effector T-cell activation or inversely reverse B cells into immunosuppressive CD55^+^ B cells for chemoresistance (41). Hence, the function of B cells seems multidirectional, and proper induction of memory B-cell activation and antibody secretion is likely necessary for immune activation.

## Complex Myeloid Cell Subsets in Breast Cancer

Myeloid cells, including neutrophils, monocytes, and macrophages, propel tumor progression, mainly *via* immunosuppression and cytokines secretion, but huge heterogeneity exits in myeloid cells (42). Macrophages are the most widespread myeloid cell group in tumor lesions and can polarize towards proinflammatory M1 or immunosuppressive M2 phenotype. M2-type genes, such as CD276, CD163, MS4A6A, and TGFB1, are widely expressed in tumor-associated macrophages and showed the characteristics of cascade M2 differentiation (32, 34, 43).

Recently, depletion of protumor macrophages with CSF1R neutralizing antibodies was shown inefficient to inhibit tumor progression (44). Sc-RNAseq identified gene sets related to M1 and M2 phenotypes, and the conversion of M2 phenotype to M1 immune-activated macrophages was an effective measure to stimulate the immune response (45, 46) and amplify ICB therapy. Moreover, a new type of lipid-associated macrophages has been found in tumors that highly express lipid metabolism genes such as Fabp5 or Apoe but not in the conventional M1/M2 classification. These macrophages also expressed PD-L1 and PD-L2 for immunoregulation (47). Considering the rich lipid sources of breast TME and the fatty acid dependence of tumor cells (48), lipid-associated macrophages may belong to the tissue-resident macrophage population that are reprogrammed by tumor cells and the metabolic drugs might be targeted.

Finally, bone marrow-derived suppressor cells (MDSCs), including polymorphonucler MDSCs (PMN-MDSCs) and monocytic MDSCs (M-MDSCs), were identified in breast tumor patients and gradually infiltrate tumor site with disease progression (27). Similar to T cells, the heterogeneity of neutrophils and monocytes could still be seen in tumors and spleen. Both tissues contained both normal mature myeloid cell populations (Camp/Lcn2/Ltf^+^ neutrophils) and immunosuppressive bone marrow-derived suppressor cells (CD84/Il1b/Spi1^+^ PMN-MDSCs). These two cell types coexist and are different from a common myeloid progenitor, proving the ability of tumor cells to modulate myeloid differentiation rather than the repolarizing mature neutrophils into CD84 and ROS high MDSC subgroup, which mainly support the immunosuppressive environment (49). In fact, compared with their role at the tumor site, neutrophils seem to play a more prominent role in tumor dissemination, metastasis, and recurrence (50–54). Circulating tumor cells (CTCs) secret cytokines CSF1, CSF3, TGF-β3, and IL-15 to recruit PMN-MDSCs to physically cluster with CTCs. Subsequently, CTC-bonded PMN-MDSCs secrete inflammatory factors TNF-α, OSM, IL-1β, and IL-6 in CTC-PMN-MDSCs niche to improve the proliferation, stress resistance of tumor cells, and CTC cluster formation (55).

## CAFs Functional Subclusters in Breast Cancer

Cancer-associated fibroblasts (CAFs) are a major component of the stroma in tumor. Healthy breast matrix is destroyed with tumor progression as healthy fibroblasts reduce and tumor cells reprogram fibroblasts to CAFs (56). CAFs are scattered inside the tumor tissue rather than in the surrounding area, relying on its strong secretion and tissue adhesion capacity. Traditional CAFs promote tumor progression mainly through stromal remodeling, immunosuppression, and neovascularization (57), but CAFs are a group of heterogeneous cells without a unified cell maker and of uncertain origin. Stromal cells such as MSCs, endothelial cells, and pericytes, are strong candidates for CAF progenitor cells (58).

Single-cell transcriptomic studies of intratumoral heterogeneous CAFs showed two prime functions including nidogen^+^ perivascular fibroblasts and fibulin^+^ stromal fibroblasts. Nidogen^+^ perivascular fibroblasts highly expressed vascular production regulators NOTCH3, EPAS1, COL18A1, and NR2F2 and were enriched for perivascular markers ACTA2, MCAM, CAV1, TAGLN, MYH11, MYLK, and RGS5, suggesting a role in the regulation of neovascularization in tumors under increased oxygen demand settings. Another fibulin^+^ stroma-related CAFs highly expressed matrix-related DCN, LUM, VCAN, LOX, secret collagens, and chemokines CXCL12 and CXCL14. These seemed to act as tumor functional fibroblasts that participate in tumor stromal formation and immune response. Interestingly, stroma-related CAFs gradually shrunk with tumor progression, which may be due to tumor space occupation, low tumor adhesion, and metastasis requirements (59, 60).

Alternatively, CAFs were divided into three functional subgroups: (1) α-SMA^+^ myofibroblasts (myCAFs) which maintain tumor structural stability and ECM remodeling, (2) inflammatory fibroblasts (iCAFs) which regulate immune response, and (3) extracellular matrix fibroblasts (ECM-CAF) which remodel the extracellular matrix of tumors. These functional cells can directly or indirectly interact with tumor cells, myeloid cells, and T cells to promote immunosuppressive milieu (61–63). Spatially, dispersed iCAFs colocalized with all lymphocyte cells, responding to chemokines (CXCL12/CXCL14-CXCR4 and CXCL10-CXCR3), complement, transforming growth factor-β (TGFB1/TGFB3-TGFBR2), and lymphocyte inhibitory/activation molecules (LTB-LTBR, TNFSF14-LTBR and LTB-CD40, VTCN1/B7H4-BTLA). In contrast, marginal myCAFs only interact directly with CD8^+^ T cells for tumor invasion and matrix remodeling (47). Interestingly, in the high-grade pregnancy-associated breast cancer, the tumor reprograms these fibroblasts into a more function-evolved phenotype. Those fibroblasts commonly express higher COL1A1, CXCL12, TGFB1, and MMP3 and have a unique fatty acid metabolism, peroxisome, and inflammatory profile (62). Therefore, the degree of malignancy of breast tumor may affect the function of CAFs.

## Breast Cancer Immunotherapy Response Microenvironment

ScRNA-seq-based supervision of the immune response following ICB is necessary to fully understand mechanism of action and expose emerging resistance pathways (64). Breast cancer patients benefiting from ICB therapy already exhibit expanded PD-1^+^ T cells before ICB therapy compared with nonresponders. Matured CD4^+^ Th1 and Tfh cells and exhausted CD8^+^ effector T cells greatly expand after anti-PD-1 therapy, with higher proliferation, immune checkpoint protein (LAG3, HAVCR2, PDCD1), effector (IFNG, NKG7), and cytotoxicity (GMZB, PRF1), higher TCR richness, and lower TCR clonality. These responsive T cells positively correlate with PD-L1^+^PD-L2^+^ DCs, PDL1^+^ CCR2^+^, or MMP9^+^ macrophages or MHC I/II^+^ cancer cells, mainly through costimulatory CD28-CD80, ICOS-ICOSLG, coinhibitory PDCD1-CD274/PDCD1LG2, HAVCR2-LGALS9, and CLTA4-CD80/CD86. In contrast, TCF7^+^ Sell^+^ naïve T cells and CX3CR1^+^ or C3^+^ macrophages are inversely correlated with T-cell expansion. More importantly, a specific gene set related to T-cell expansion has been identified for the prediction of ICB response (65). Similar to the functional B cells in chemotherapy (41), the abundant B-cell population in breast tumors are activated by antigen presentation after ICB therapy to promote T follicular helper cell expansion and cytotoxic CD8^+^ T cells for immunotherapy sensitization (40), which recalls breast TME into “immune hot” microenvironment.

## Spatial Distribution of Breast TME

Besides the heterogeneity and TCR/BCR clonal evolution of stromal and immune cells, tumor histological regions are demarcated by TME to form heterogeneous tumor organization (30). However, single-cell dissociation destroys the intact tissue structure in scRNA-seq (33) and cell location and orientation information were lost during tissue dissociation. Therefore, tremendous effort has been put into the development of a variety of a three-dimensional (3D) near-realistic cell environment. Spatial transcriptome analysis and single-cell-level depth of tumor pathology (66) was developed to evaluate cell types and their locations. In 2018, Keren et al. first used multiplexed ion beam imaging by time-of-flight (MIBI-TOF) to simultaneously analyze 36 proteins at single-cell resolution and draw a rough single-cell map of breast cancer immune distribution. This study highlighted that function-similar immunoregulator cells cluster together for stronger effects. For example, KI67^+^ proliferating cells or IDO^+^ immunoregulatory cells formed proliferated or immunoregulatory units within the tumor. Moreover, tumors were divided into three immune categories: (1) “cold tumors” with less immune cell infiltration, (2) “compartmentalized tumors” where immune cells were distributed around tumor cells in an organized regional orientation, and CD4^+^ T cells were mainly near PD-1^+^ cells in the tumor-immune border, and (3) “mixed tumors” with tumor cells and immune cells mixed together without borders, where CD8^+^ T cells were dominant and had relative stronger immunosuppression with worse prognosis (12).

Based on stromal distribution, topological network of high-dimensional single-cell mass cytometry images, tumors were compartmentalized in: (1) low stromal environment (including immune cell infiltration and non-TME cell infiltration), (2) highly vascularized regions, (3) vimentin high fibroblasts regions, (4) fibronectin high fibroblasts regions, and (5) multicellular dispersive types, where fibroblasts are distributed around tumor cells and blood vessels which are consistent with previous observation (59). Meanwhile Ki67^+^ tumor cells and T-cell infiltration were increased around the blood vessels.

The traditional PAM50 is too generic to reveal the tumor composition within individual patients. Based on tumor cell metacluster compositions and environmental interaction, tumor lesions can be divided into 18 single-cell pathology subgroups (SPCs), and then matched with stromal models to establish 11 stromal environment subgroups. Interestingly, these immunological patterns and pathological subtypes were correlated with overall survival. For example, hypoxic SPC17 TNBC often exhibited large, stroma-deficient tumor regions, whereas SPC 13-16 TNBC showed T-cell-enriched or macrophage-enriched regions, and HR^+^ tumor was immune cold, accompanied by a range of fibroblast-enriched stromal environment (67). For now, the basic elementary interaction between tumor cells and TME have been explored, but more complex interactions with specific tumor regions and cell subtypes still need further elucidation.

**Figure 2:**
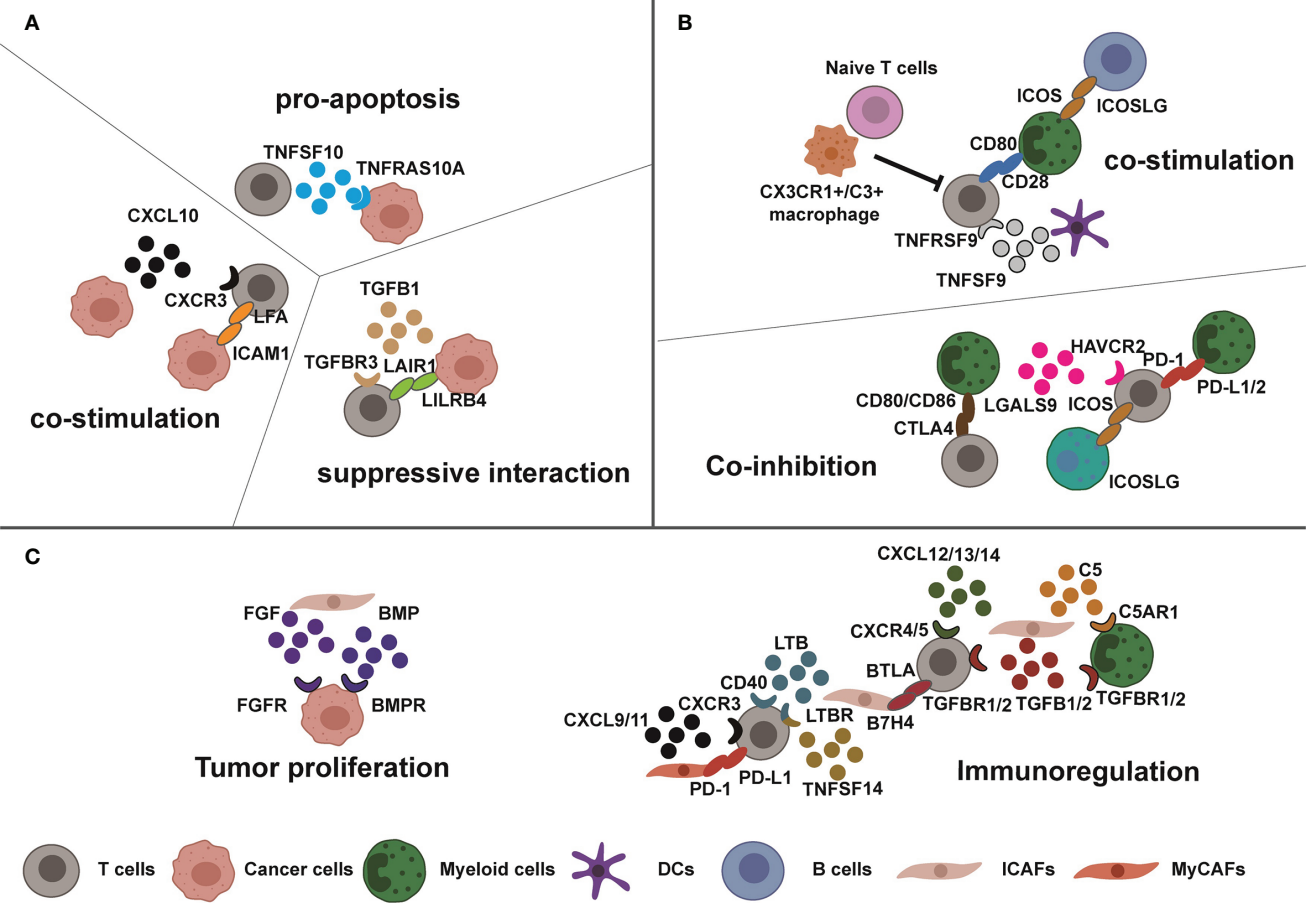
Complex interactions in breast tumor microenvironment. Tumor cells, immune cells, and CAFs exhibit high interactions which dynamically change cellular functions. **(A, B)** Sometimes, the functions of these interactions are opposite such as the costimulatory or suppressive interaction between T cells and tumor cells or myeloid cells due to specific tumor homeostasis. **(C)** Meanwhile, the CAFs interact with T cells, cancer cells, and myeloid cells for tumor progression and immunosuppression through cytokines and immunomodulatory proteins.

High-resolution spatial transcriptomics can distinguish hundreds of different spots in the tissue (68); each containing tens of cells which expression profile can be deconvoluted with RNA sequencing. Coupled with the resolution of fluorescence *in situ* hybridization (FISH) and *in situ* sequencing (ISS), spatial transcriptome analysis of intact tissue sections can expose spatial nanoscale-resolution imaging (68, 69). When combined with sc-RNAseq and spatial transcriptome positioning on breast cancer patients, this technique confirmed the aforementioned cell heterogeneity and explored the interaction within specific functional TME cells (**Figure 2**). Primary tumors were first divided into 9 different gene regulatory pattern regions based on tumor biology and TME components (tumor ecotypes). Some ecotypes enriched basal subtype classification, cycling and luminal progenitor cells, and few immune cell infiltrations, which corresponded to very poor 5-year survival. Another highly immune cell-infiltrating ecotype is represented by a higher response to ICB therapy (47).

Recently, deep learning convolutional algorithms were established to directly learn single-cell spatial transcriptomes and matched them to any HE-stained tissue section. These can be used for tumor and TME cell model diagnosis prediction (70). Hopefully, after accumulating enough spatial transcriptome patterns, clinical pathological deep learning algorithm-based HE images can directly recognize accurate tumor subtypes and make more precise therapeutic interventions.

## Outlook

The current single-cell omics results have drawn a relatively detailed map of the breast TME, distinguished stromal cells and immune cells into functional populations (**Table 1**), and greatly enriched the TME components for clinical diagnosis and targeted therapy intervention. Unfortunately, many more in-depth technical methods and joint application methods have not yet fully matured, since single-cell omics only just emerged. The microenvironment related to the occurrence and development of breast cancer has not been fully researched.

1. Some cell populations in tumors such as NK cells, neutrophils, and DCs have not been fully understood,
2. The microenvironment composition is still unclear in the tumor initiation, dissemination, and metastasis. Macrophages are necessary for early breast tumorigenesis (71), but single-cell transcriptome can hardly identify the microenvironment composition of early tumorigenesis due to the difficulty to capture small lesions and cell number limitations.
3. The current microenvironment recognition method is mainly established by single-cell transcriptome and antibody-dependent multidimensional imaging. However, immune cells are functionally regulated by numerous transcription factors (21), hence, other single-cell strategies such as scATAC-seq, scChIP-seq, and the emerging CRISPR screening (72, 73) will be useful for breast TME analysis.
4. TME analysis needs to pay more attention to the combination of cell function and spatial distribution in the future. For example, metabolic deprivation of polytrophic tumor cells transforms the shared TME metabolism dependence (74, 75); whereas, various TME cells have different substrate dependencies for its activation or immunosuppression, such as the correlation between TCA cycle and phenotypic states of T cells (32), high lipid metabolism in lipid-related macrophages (47), and high fatty acid metabolism in function-evolved CAFs (62). However, there is still no systematic research on breast TME immunometabolism. Indeed, combining scRNA-seq and spatial transcriptomics enables detailed analysis of cellular metabolic pathways as well as regional metabolic pathway enrichment. Moreover, the combination of spatial transcriptome and spatial metabolome (76, 77) will clarify the metabolic status in indicated tumor lesions. Using spatial metabolome to analyze TME metabolic pathways, combined with spatial transcriptome diagnosis of TME cell subtypes, can accurately detect the metabolite preference of TME cells. Further research is needed to explore these methods in detail.
5. Similarly, single-cell proteomics has not yet been completed (78). Current scLC-MS-based proteomics technology SCoPE-MS and NanoPOTs sample preparation technology can only quantify ~1,000 proteins per cell across thousands of individual cells (79–81), not to say the detailed biological analysis, carrier proteome, and data standardization (82–84).
6. Deep learning-based neural networks may identify novel breast cancer subtypes, applying more cost-effective and rapid single-cell analysis to clinical tumor patients. This will strengthen pathological diagnosis and therapeutic intervention optimization.

**Table 1:** Breast TME is heterogenous with various cell subtypes.

Cell type	Subtype	Makers	Characteristic	Reference
**CD4^+^ T cells**	Naïve CD4^+^ T cell	TCF7^+^, Sell^+^	Negative correlation with CD4^+^ effector T cells	(26, 31–35)
	CD4^+^ Tem	CD44^+^, ANXA^+^	Enrichment with effector function, proinflammation, immune cell homing, antigen presentation, and immune checkpoint; Activating with IFN, hypoxia, TCA cycle, and TCR; higher IC expression indicated ICB response	
	CD4^+^ Tcm	CCR7^+^		
	Tfh	CXCL13^+^		
	Th1	IL7R^+^		
	Treg	FOXP3^+^	Immunosuppression	
**CD8^+^ T cells**	Naïve CD8^+^ T cell	TCF7^+^, Sell^+^	Negative correlation with CD8^+^ effector T cells	
	CD8^+^ Tem	GZMK^+^, STMN1^+^	Enrichment with effector function, proinflammation, immune cell homing, antigen presentation, and immune checkpoint; Activating with IFN, hypoxia, TCA cycle, and TCR; higher IC expression indicated ICB response	
	CD8^+^ Tcm	GZMK^+^, GZMA^+^		
	CD8^+^ Trm	GZMB^+^, CCL3^+^		
	CD8^+^ CD103^+^ T cell	CD103^+^		
**B cells**	Naïve B cell	IGHM high	More memory B cells in primary tumor increased BCR diversity in primary tumor; Secreting antibody and activating T cells for ICB response; ICOSL^+^ B cells activate T cells in response to chemotherapy	(36, 40, 41)
	IGM^+^ CD27^+^ memory B cell	IGM^+^, CD27^+^		
	CD27^−^ atypical memory B cell	IGM^+^, CD27^+^		
	Class-switched memory B cell	AICDA^+^, IGHG^+^		
	Plasma cell	CD27^+^, CD38^+^		
	CD14^+^ atypical B cell	CD14^+^		
	Germinal center B cell	CD38^+^, BCL6 high		
**Macrophages**	M1	CX3CR1^+^, C3^+^	Proinflammation function	(32, 34, 43, 45, 47)
	M2	PDL1/2^+^, CD163^+^, MS4A6A^+^	Playing immunosuppressive function with the expression of PDL-1/2, CXCL9/10, CCL8	
	Lipid-associated macrophage	FABP5^+^, APOE^+^	Higher lipid metabolism with the expression of PD-L1 and PD-L2	
**Neutrophils**	Mature neutrophil	CAMP^+^, LCN2^+^, LTF^+^	Similar with normal neutrophil	(27, 49, 55)
	PMN-MDSC	CD84^+^, IL1, SPI1^+^	Immunosuppressive function, interact with CTCs by TNF-α, OSM, IL-1β, and IL-6	
**Monocytes**	Mature monocyte	CD84^−^, LY6C^+^	Similar with normal monocyte	
	M-MDSC	CD84^+^, LY6C^+^	Immunosuppressive function	
**CAFs**	My-CAF	ACTA2, MYLK^+^, MYH11^+^	ECM remodeling, vascularization	(47, 59–63)
	I-CAF	LY6C1^+^, C3^+^, C4B^+^	Immunomodulation and chemokines secretion	
	ECM-CAF	TNC^+^, COL18A1^+^, COL12A1^+^	ECM remodeling	
	Involuted CAF	COL1A1^+^, CXCL12^+^, MMP3^+^	Higher immunomodulation and ECM remodeling, only exited in pregnancy-related breast cancer	

In summary, the evolution of novel single-cell omics technology, including abundant omics exploration strategies, multiomics conjoint analysis mode, and deep learning network architecture is still developing and has the potential to revolutionize our understanding of the TME, its changes with disease progression, and its response to therapy.

Here, we focused on the applications and advances of single-cell omics to unveil the heterogeneity, pathogenesis, and treatment of breast cancer, describing the complex model of breast cancer microenvironment composition in detail. Despite the massive heterogeneity between breast cancer patients and tumors, the components of the immune microenvironment seem to reflect the patient’s survival and response to immunotherapy, highlighting the importance of fully understanding TME changes and progression.

## Author Contributions

HZ and ZT conceived and conducted the project. HZ and SW supervised the project. ZT and HZ wrote the paper. ZT, CK, FY, MW, and MS performed the review organization and analysis. MW contributed to manuscript editing. All authors listed have made a substantial, direct, and intellectual contribution to the work and approved it for publication.

## Funding

This work was supported by the National Natural Science Foundation of China (reference numbers 81670097, 81870085, and 81273004), Grants for Scientific Research Enhancement of Anhui Medical University (2019xkjT004 and XJ2020019), and Grants for Collaborative Innovation Project of Colleges and Universities in Anhui Province (GXXT-2021-063).

## Conflict of Interest

The authors declare that the research was conducted in the absence of any commercial or financial relationships that could be construed as a potential conflict of interest.

## Publisher’s Note

All claims expressed in this article are solely those of the authors and do not necessarily represent those of their affiliated organizations, or those of the publisher, the editors and the reviewers. Any product that may be evaluated in this article, or claim that may be made by its manufacturer, is not guaranteed or endorsed by the publisher.
